# Detection of pathogens associated with acute febrile illness in children under five years of age in rural Tanzania

**DOI:** 10.1038/s41598-025-96190-5

**Published:** 2025-04-04

**Authors:** Athanasia Maro, AbdulHamid S. Lukambagire, Blandina T. Mmbaga, Jie Liu, Sixbert I. Mkumbaye, Nelson Amani, Judith Njau, Reginald A. Kavishe, Jean Gratz, Eric R. Houpt, Jo E. B. Halliday

**Affiliations:** 1 KCMC University, Moshi, Tanzania; 2 Kilimanjaro Clinical Research Institute, Moshi, Tanzania; 3 Paediatric and Child Health Department, Kilimanjaro Christian Medical Center, Moshi, Tanzania; 4 School of Public Health, Qingdao University, Qingdao, China; 5 Division of Infectious Diseases and International Health, University of Virginia, Charlottesville, VA USA; 6 School of Biodiversity, One Health and Veterinary Medicine, College of Medical, Veterinary and Life Sciences, University of Glasgow, Glasgow, G12 8QQ UK

**Keywords:** Acute febrile illness, TaqMan array card, Non-malaria fever, Real-time PCR, Tanzania, Zoonoses, Epidemiology, Infectious diseases

## Abstract

Acute febrile illness (AFI) investigations are crucial for public health. They can provide data on disease prevalence, morbidity, and mortality, and improve treatment, management, control, and detection of outbreaks in areas with limited diagnostic tests. Current understanding of multiple causes of AFI in the paediatric population in Tanzania is limited. This study aimed to simultaneously detect 33 pathogens using TaqMan Array Card based real-time PCR. Whole blood samples were collected from a total of 247 children (2–59 months old) who presented with febrile illness at Dareda and Haydom hospitals in north-eastern Tanzania between November 2015 and March 2016. Overall, 50 (20.2%) and 8 (3.2%) of 247 children had at least one and more than one pathogen detected respectively. Bacterial zoonoses were frequently detected including *Brucella* spp. (*n* = 18, 7.3%), *C. burnetii* (*n* = 4, 1.6%), *Bartonella* spp. (*n* = 3, 1.2%), *Rickettsia* spp. (*n* = 3, 1.2%) and *Leptospira* spp. (*n* = 1, 0.4%). Dengue virus was detected in 14 (5.7%) individuals and *Plasmodium* spp. in 12 (4.9%) individuals. These findings reveal the potential clinical importance of zoonoses and arboviruses in febrile children in Tanzania and highlight the need to consider a broad range of pathogens in febrile illness diagnosis.

## Introduction

Acute febrile illness (AFI) is a leading cause of presentation at primary health facilities in the tropics ^1^ that contributes to high burdens of morbidity and mortality in sub-Saharan Africa and worldwide ^2,3^. Several infectious agents including viruses, parasites, and fungi can cause AFI, as well as non-infectious conditions like autoimmune diseases, inflammatory disorders, cancer, and drug reactions ^4^. The majority of deaths in children younger than five years (64.0%) are attributed to infectious causes ^5^. The infectious causes of AFI vary widely by age groups, immune status, geographic locations, ecology, and climate ^6,7^. Differential diagnoses are made by considering clinical presentations, patient history, results from diagnostic tests, and epidemiological factors. However, diagnosing acute febrile illness can be challenging because many diseases can present with similar symptoms and diagnostic tools for the full range of potential causes are often lacking ^3,8,9^.

AFI aetiology studies are crucial to inform empirical treatment and care, for generating estimates of prevalence, morbidity, death, and economic effect of multiple pathogens and to inform public health intervention development. Studies generating data on multiple pathogens can also detect novel and re-emerging pathogens and identify new outbreaks in contexts where there are few laboratory diagnostic tests available to identify causes of AFI ^8^.

TaqMan array cards (TAC) have been developed to enable simultaneous detection of multiple pathogens ^10^. TAC is a real-time PCR based system that is sensitive and specific for multiple pathogen detection and has been used for surveillance, emerging and re-emerging pathogen detection, and outbreak investigation in high and low malaria endemic contexts in Sub-Saharan Africa ^9–15^.

In Tanzania, there has been extensive research on AFI but the majority of studies have focused on individual pathogens. Only a small number of studies in Tanzania have included multi-pathogen diagnostic approaches ^9,14,16–19^. Of these, most have enrolled patients of all ages and provide data on AFI in adults and adolescents predominantly. There remains a lack of data on AFI aetiology in Tanzania overall and specifically in children under 5 years. This study aimed to simultaneously detect 33 agents that are associated with AFI in children under five years of age who sought medical care at Haydom and Dareda hospitals in Manyara Region, north-eastern Tanzania.

## Methods

### Study design, population, and sampling

A cross-sectional, hospital-based study was conducted to enrol children presenting for care with febrile illness to the outpatient or inpatient departments of Dareda and Haydom hospitals in the Manyara region of north-eastern Tanzania. These hospitals provide a range of healthcare services and serve predominantly agro-pastoral communities ^20^ in an area of low malaria endemicity ^21^.

Children aged 2–59 months with an axillary temperature of ≥ 38°C at the time of presentation were eligible for inclusion in the study. Children receiving anti-retroviral therapy, tuberculosis therapy, or who reported use of antibiotic therapies for three or more days in the two weeks before screening were excluded. Whole blood samples of up to 2.5mL volume were collected into EDTA vacutainers at the time of enrolment, stored temporarily at 2–8°C for up to three days then stored at -80°C, shipped to the Kilimanjaro Clinical Research Institute Biotechnology Laboratory and stored there at -80 °C freezer until TAC testing.

Demographic and socio-economic data recorded for study participants included participant age in months, sex, the occupation of their mother and father, and if domestic animals were kept in their household. Clinical data recorded (for patients presenting to Haydom only) included the final diagnosis recorded in hospital records. Given variable information available to clinicians to inform this assessment and variable timing of field recording after initial presentation analyses of these data were limited to checks of the mention (or not) of pathogens (and corresponding disease names) detected in 10 or more individuals through TAC testing in the clinical diagnosis field.

### Nucleic acid extraction and TaqMan array card testing

High pure viral nucleic acid large-volume kits (Roche, Germany) were used for nucleic acid extraction as per the manufacturer’s instructions. Briefly, 1.5 mL of EDTA blood was subjected to a lysate preparation process, followed by purification and elution of nucleic acid using spin columns, including a high pure extender assembly for an initial large volume. Extrinsic controls PhHV (Phocine Herpesvirus) for DNA targets and MS2 bacterial phage for RNA targets were added to each sample during the lysate preparation to evaluate extraction and amplification efficiency. For each batch of extractions, a blank was processed through the complete protocol and later tested on a real-time PCR to rule out contamination. The eluted nucleic acids were stored at 80 °C before testing.

A TAC system as described previously ^10^ was used to simultaneously detect 33 AFI-associated viral, bacterial, and parasitic pathogens. Target pathogens were Chikungunya virus, Dengue virus, Zika virus, Crimean Congo Haemorrhagic Fever (CCHF) virus, Bundibugyo virus, Ebola virus, Sudan virus, Hepatitis E virus, Lassa virus, Marburg virus, Measles virus, Nipah virus, O’nyong-nyong virus, Rift Valley fever virus, West Nile virus, Yellow fever virus, *B. anthracis*,* Bartonella* spp., *Brucella* spp., *C. burnetii*,* Leptospira* spp., *N. meningitidis*,* Rickettsia* spp., *S. enterica* Typhi, *S. enterica* Paratyphi A, *S. aureus*,* S. pneumonia*, Group A *streptococcus*,* Y. pestis*,* Leishmania* spp., *Plasmodium* spp., and *T. brucei.* Primers and probes at concentrations of 900 nM and 250 nM respectively were used ^10^. Test cards were loaded with a mixture of 75 µl of total nucleic acid and 25 µl of TaqMan fast virus one-step mastermix (Life Technologies; Thermo Fisher Scientific, Carlsbad, CA). No template, positive controls (for all target pathogens), and extrinsic controls (PhHV and MS2) were included in all test runs. Cards were run on the ViiA7 real-time PCR system (Life Technologies, Thermo Fisher Scientific, Carlsbad, CA) using the following PCR cycling conditions: reverse transcription step of 10 min at 50 °C and 1 denaturation cycle of 20 s at 95 °C followed by 40 two-step cycles of 3 s at 95 °C and 30 s at 60 °C. A run was considered valid if all positive controls were amplified, extrinsic controls were amplified for all samples, and no negative controls amplified. A sample was considered positive for a given target when it showed amplification with a cycle threshold (Ct) < 35. Samples showing amplification for a given target with Ct values ≥ 35 were considered borderline and were re-tested in a singleplex qPCR on the ViiA7 instrument using the individual target primers and probes ^10^ and identical cycling conditions. Samples with Ct < 40 in these confirmatory singleplex reactions were considered positive. Samples where amplification was not confirmed in the singleplex reaction were classified as negative. All other samples were classified as negative. All samples classified as positive and borderline for *T.brucei* in the TAC assay were re-tested in singleplex reactions and classified based on these singleplex reactions.

Samples that were *Brucella* spp. positive were additionally tested by quantitative PCR assay to distinguish between *B. melitensis* and *B. abortus* DNA. The *Brucella* speciation assay applied previously published primers, probes, and procedure ^22^. Briefly, duplicate reactions were performed on the Rotorgene platform (Qiagen) in 25 µL volumes, containing 25 µL TaqMan Universal Master Mix, 0.2 µM of each primer, 0.1 µM of each probe and 2.5 µL of template nucleic acid. Cycling conditions were as previously described (10 min at 95 °C followed by 45 cycles at 95 °C for 15 s and 57 °C for 1 min). Positive (*B. abortus* 544 and *B. melitensis* 16 M DNA) and negative (no template) controls were included in these assays. Samples were classified as positive for *B. abortus* and *B. melitensis* if a Ct < 40 was observed in either test well.

### Data analysis

All data analyses, summaries, and graphs were performed using R version 4.3.2 ^23^. Continuous variables were summarized using median and interquartile range (IQR). Categorical variables were summarized as frequencies and percentages. Binomial confidence intervals were calculated for all proportions.

### Ethics declarations

This research was performed in accordance with relevant guidelines and regulations. Written consent was obtained from the parents or guardians of all participants before enrolment in the study. Ethical clearance for the collection and storage of participant samples was obtained from the College Research Ethics Review Committee (CRERC) of Kilimanjaro Christian Medical University College, certificate number 835. Additional clearance for testing for AFI pathogens was approved by the CRERC, certificate number PG 01/2022 and by the Medical Research National Health Research Ethics Review Committee (NIMR/HQ/R.8a/V0l.1X/2079).

## Results

### Socio-demographic distribution of participants

A total of 247 blood samples were obtained from febrile children who were recruited at Dareda and Haydom hospitals from November 2015 to March 2016. The demographic and clinical characteristics of these individuals are shown in Table 1. The majority of household respondents (82.2%) reported keeping domestic animals and farming was a dominant occupation for both mothers and fathers of the enrolled children (Table 1).

**Table 1:** Socio-demographic and clinical characteristics of participants who attended Haydom and Dareda hospitals in 2015–2016 (*N* = 247).

Variable	Level	Median (range)	*n*	%
Age (months)		19 (2–59)		
Gender				
	Male		108	43.7
	Female		139	56.3
Mother ‘s Occupation				
	Farmer		219	88.7
	Employed		28	11.3
Father’s Occupation				
	Farmer		162	68.1
	Employed		76	31.9
	NA		9	-
Domestic animals at household				
	Yes		203	82.2
	No		44	17.8
Brucellosis in clinical diagnosis				
	Yes		0	0
	No		133	100
	NA		114	-
Malaria in clinical diagnosis				
	Yes		17	12.8
	No		116	87.2
	NA		114	-
Dengue in clinical diagnosis				
	Yes		0	0
	No		133	100
	NA		114	-

### Pathogens detected by TAC real-time PCR

**Fig. 1:**
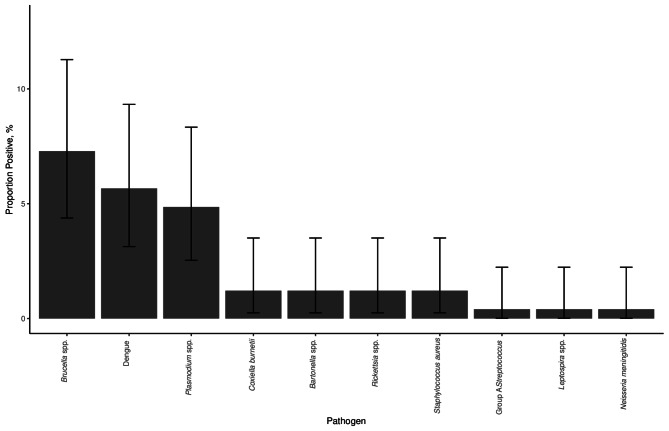
Proportion of individuals testing positive for pathogens detected by TAC in a population of febrile children (≤ 5 years) presenting to hospitals in Haydom and Dareda, northern Tanzania, 2015–2016 (*N* = 247). Bars show exact binomial confidence intervals.

**Fig. 2:**
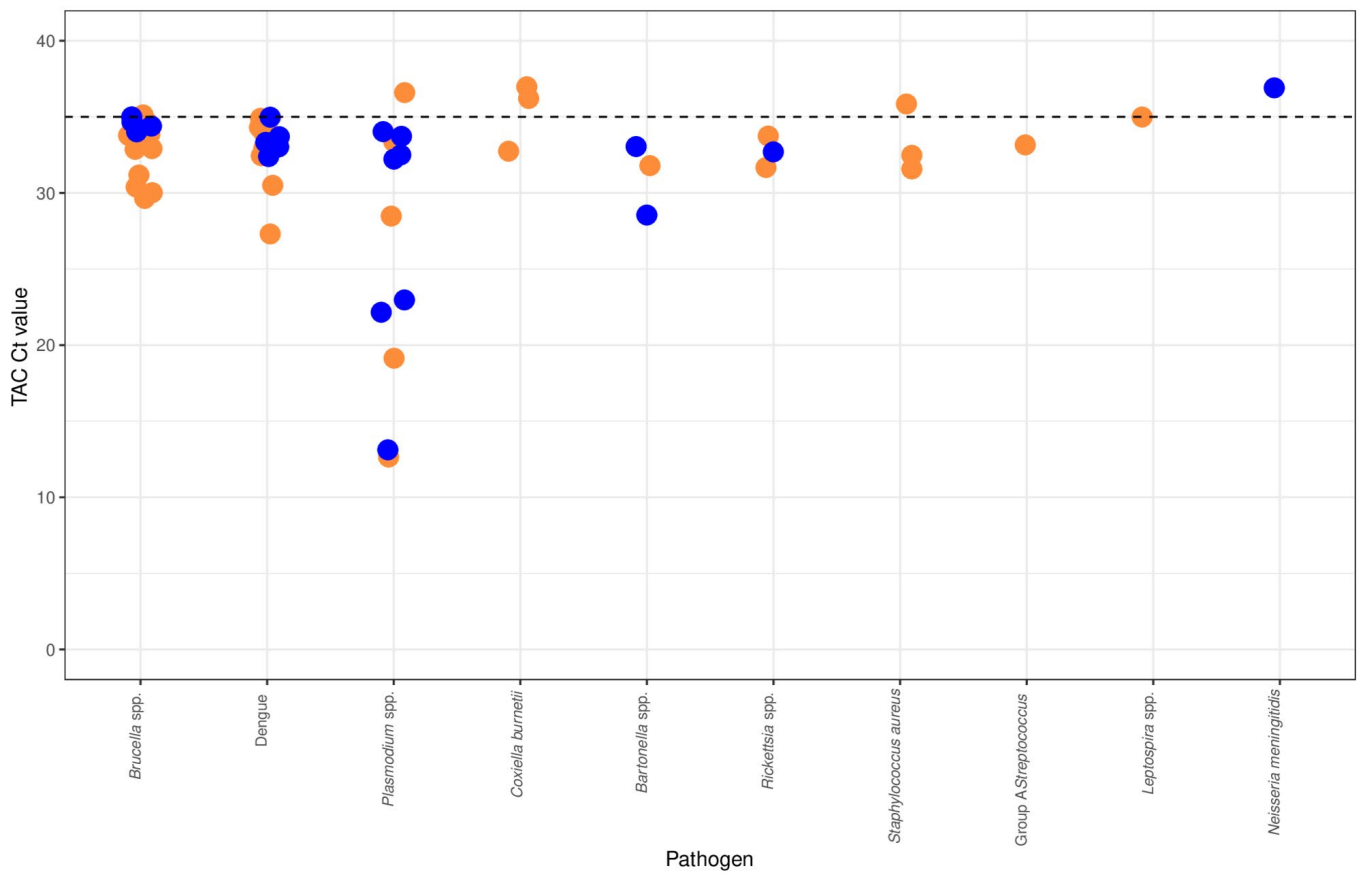
Cycle threshold (Ct) values observed for the pathogens detected by TAC in a population of febrile children (≤ 5 years, *N* = 247), presenting to hospitals in Haydom (orange points) and Dareda (blue points), northern Tanzania, 2015–2016. The dashed line at Ct = 35 indicates the threshold applied to differentiate samples classified as positive (Ct < 35) and borderline (Ct ≥ 35) by TAC.

Of the 247 individuals tested, 50 (20.2%) individuals had one or more pathogens detected, including 8 bacteria, 1 protozoan, and 1 virus. The number and proportion of individuals positive for each pathogen is shown in Table 2; Fig. 1. The Ct values seen for each pathogen detected by TAC are shown in Fig. 2. A total of 8 (3.2%) individuals were positive for more than one pathogen. One individual had positive TAC results for three pathogens: *C. burnetii*, dengue, and group A *Streptococcus*. Seven individuals had two pathogens detected, including dengue and *Brucella* spp. (4 individuals), dengue and *Rickettsia* spp. (1 individual), *Brucella* spp. and *S. aureus* (1 individual), and *Bartonella* spp and *Plasmodium* (1 individual).

**Table 2:** Pathogens detected by TAC from children presenting for care with AFI at Haydom and Dareda hospitals in Tanzania, 2015–2016 (*N* = 247).

Pathogen	Haydom *N* = 131, *n* (%)	Dareda *N* = 116, *n* (%)	Total *N* = 247, *n* (%)
*Brucella* spp.	13 (10.0)	5 (4.3)	18 (7.3)
Dengue virus	9 (6.9)	5 (4.3)	14 (5.7)
*Plasmodium* spp.	5 (3.8)	7 (6.0)	12 (4.9)
*Bartonella* spp.	1 (0.8)	2 (1.7)	3 (1.2)
*Coxiella burnetii*	3 (2.3)	0 (0)	3 (1.2)
*Rickettsia* spp.	2 (1.5)	1 (0.9)	3 (1.2)
*Staphylococcus aureus*	3 (2.3)	0 (0)	3 (1.2)
Group A *streptococcus*	1 (0.8	0 (0)	1 (0.4)
*Leptospira* spp.	1 (0.8)	0 (0)	1 (0.4)
*N. meningitidis*	0 (0)	1 (0.9)	1 (0.4)

### *T. brucei* singleplex and *Brucella* speciation real-time PCR

None of the three samples initially positive for *T. brucei* by TAC were positive in the singleplex confirmatory test, so all were classified as negative for *T. brucei*. Of the 18 samples that were TAC positive for *Brucella* spp. five also amplified with *B. abortus*-specific primers. None amplified with *B. melitensis*-specific primers.

### Clinical diagnosis

Clinical diagnoses were only recorded from participants enrolled in Haydom. Of the 133 enrolled in Haydom, 17 (12.8%) had malaria included in their clinical diagnosis. A range of pathogens were detected by TAC in this population of individuals with malaria included in the clinical diagnosis. One individual with a clinical diagnosis of malaria was TAC positive for *Plasmodium.* Other pathogens detected in this population of 17 individuals were *Brucella* spp. (*n* = 1), *Coxiella burnetii* (*n* = 1), dengue (*n* = 2), group A *Streptococcus* (*n* = 1) and *Rickettsia* spp. (*n* = 2). Neither brucellosis nor dengue were mentioned in the clinical diagnosis for any patients presenting to Haydom (Table 1).

## Discussion

This study used TAC technology to detect eleven different pathogens associated with AFI in whole blood samples collected from children aged 2–59 months presenting with febrile illness from the rural agro-pastoral community of Manyara region in north-eastern Tanzania. Malaria was detected less frequently (*n* = 12 detections) than non-malaria pathogens combined (*n* = 45 detections). The two pathogens detected at highest frequency were *Brucella* spp. and dengue virus, both of which were detected more frequently than *Plasmodium* (Table 2; Fig. 1).

The overall proportion of children in whom one or more pathogens were detected by TAC was 20.2% in this study. This proportion is similar to the findings of a previous study using comparable blood TAC PCR diagnostics in Zanzibar (18.2%) ^14^. However, a similar study conducted in a high malaria endemicity region of Kilombero, Tanzania, found overall blood TAC PCR positivity of 49% in individuals aged one year and above ^9^. A study in Burkina Faso, Madagascar, and Sudan that used blood TAC with an expanded set of pathogens detected one or more pathogens in 62%, 24%, and 60% of febrile children and adolescents in these countries respectively ^15^. Although the target populations, methods for sampling and diagnostics are not all directly comparable, the consistent findings across these studies, that are also seen in this Tanzanian population, are the identification of zoonotic bacteria and arbovirus as prevalent pathogens among febrile patients ^14,16,19^.

Bacterial pathogens dominate the pathogens identified in this population and the majority of pathogens identified are zoonoses. Of the bacterial pathogens, *Brucella* spp. was detected in 7.3% of the participants. Brucellosis diagnosis is very challenging, with confirmed brucellosis defined by culture positivity or demonstration of seroconversion in paired samples collected several weeks apart ^24^. These tools are rarely available in many brucellosis endemic contexts. Many serology based tests for brucellosis that are widely used in East Africa have very low accuracy ^25^ and consequently, brucellosis cases are often misdiagnosed. The proportion of febrile children with *Brucella* spp. detection identified in this study (6.9%) is comparable to the prevalence of acute brucellosis (6.1%) identified using culture and serological testing in a predominantly pastoralist population of children and adults sampled in northeastern Tanzania in 2016–2017 ^26^. A study of febrile children and adults performed in north-eastern Kenya in 2011 classified 15.4% of individuals as probable brucellosis cases based on RT-PCR - based testing ^27^. Similarly, PCR based testing identified *Brucella* spp DNA in 27.6% individuals aged ≤ 20 years olds with clinically suspected brucellosis sampled at two predominantly pastoralist sites in Kenya ^28^. In livestock-keeping populations with endemic livestock brucellosis, children account for high proportions of acute cases ^29^. The interpretation of *Brucella* spp. DNA detection in human samples and use of DNA detection to define brucellosis disease status in endemic contexts is a topic of ongoing research. Test positivity based on PCR detection can persist for long periods after initial infection and is not always associated with active disease ^30^. However, in this population of under five year old children these detections are more likely associated with acute infections. The *Brucella* speciation assay revealed that five out of 18 individuals who were positive for *Brucella* spp had *B. abortus* detected while there were no detections for *B. melitensis*. The likely higher sensitivity of the TAC *Brucella* assay, which targets a multi-copy gene and the species-specific assays, which target single copy genes, may explain the low proportion of TAC positives that were *Brucella* species confirmed. Further investigation of the relative performance of these assays is needed. *B. abortus* and *B. melitensis* are typically associated with cattle or sheep and goats respectively and human infections are typically more severe with *B. melitensis* ^29^. Human brucellosis is typically acquired through close contact with livestock (cattle, sheep, and goats) or consumption of raw animal products ^29^. Consumption of purchased milk was associated with increased risk and boiling milk was associated with reduced risk of *Brucella* spp. DNA detection in a Kenyan population of febrile individuals ^27^. Infection via consumption of unpasteurized cattle milk or through contact with livestock in the peri-domestic environment may explain the prevalence of *Brucella* spp. DNA detection in this population of young children.

Dengue fever is a globally important mosquito-borne viral disease of considerable public health concern. Dengue virus is endemic and re-emerging in tropical and subtropical countries ^31,32^. Globally, severe dengue is more likely in children ^33^. Since 2010 Tanzania has experienced multiple outbreaks reported every two years in Dar es Salaam and coastal regions ^34,35^. Despite the favourable climate for *Aedes* mosquitoes there are limited data on dengue in paediatric populations in north-eastern Tanzania. In this study, blood TAC PCR has detected dengue virus in 5.7% of 247 febrile children. This finding is important as no previous studies have detected dengue at this prevalence in Tanzania. A fever study in the neighbouring Kilosa region detected active dengue infection at higher prevalence in children of five years of age and above than those below five years ^36^. These findings highlight the importance of using the TAC real-time PCR system. Real-time PCR is a sensitive and specific diagnostic tool that can detect acute phase viral infections ^37^. The identification of the dengue virus in this paediatric population suggests that dengue might be an under-recognized cause of fever in Tanzania. This underscores the need to increase awareness and improve diagnostic practices among healthcare providers. It is also important to understand the true burden and transmission dynamics of dengue in different age groups within the Tanzanian population through strengthening surveillance and epidemiological studies.

This study found that 4.9% of febrile children had *Plasmodium* spp. detected. The prevalence obtained from this study using real-time PCR is higher than expected based on findings of the Tanzania demographic health malaria indicator survey that malaria prevalence in children of less than five years of age has been reduced to < 1% in Manyara region since 2016 ^38^. Comparing the TAC results for *Plasmodium* spp. with the clinical classification of the febrile children at Haydom only one individual with a clinical diagnosis of malaria was TAC positive for *Plasmodium.* The higher prevalence detected by TAC may indicate enhanced detection of asymptomatic or subclinical malaria. Previous research indicates poor correspondence between clinical and laboratory-based diagnoses of malaria^16^ and that the use of sensitive PCR diagnostic tests may overestimate the rate of undiagnosed malaria ^39^.

This study has some limitations. TAC cards were used to detect 33 pathogens, and although a wide range of AFI pathogens was explored, not all infections known to cause fever in this or similar populations were fully covered. AFI studies conducted elsewhere have included a greater variety of pathogens and revealed pathogens at high prevalence, such as a recent AFI study in Senegal that highlights the role of *Borrelia* spp. in AFI ^40^. The blood samples used in this study had been stored for several years. Although samples were stored at -80 °C and a sensitive detection technique was used, there is the possibility that some nucleic acid might have been degraded leading to a lack of detection or higher detection Ct values than would have been observed if samples were tested immediately. The exclusion of children who reported using antibiotic therapies for three or more days in the two weeks before screening may have resulted in error in estimates of the prevalence of several bacterial pathogens in the tested population compared to the total population seeking care. Due to the lack of control participants and limited clinical outcome data for this population, it is challenging to determine the clinical relevance of pathogen detections and to make inferences about the clinical impact of the detected infections.

## Conclusion

Findings from this study suggest that dengue virus and zoonoses such as brucellosis, bartonellosis, Q fever, rickettsiosis, and leptospirosis are important causes of acute febrile illness in children presenting from agro-pastoral communities in Manyara north-eastern Tanzania. Testing for several aetiologies of fever can help inform development of fever management and treatment guidance aimed at improving patient outcomes. Utilizing the TaqMan Array Card Real-time PCR systems for periodic surveillance provides a powerful and efficient tool for the detection of pathogens in diverse samples, contributing to early detection of pathogens, identification of context specific pathogen burdens, development of effective responses targeting multiple pathogens, and integrated health monitoring.

## Data Availability

The datasets generated and analysed for this study are available in the Enlighten research data repository of the University of Glasgow - https://doi.org/10.5525/gla.researchdata.1700.
